# Prevalence of Asymptomatic Parasitemia and Gametocytemia in HIV-Infected Children on Differing Antiretroviral Therapy

**DOI:** 10.4269/ajtmh.17-0462

**Published:** 2017-11-20

**Authors:** Charlotte V. Hobbs, Erin E. Gabriel, Portia Kamthunzi, Gerald Tegha, Jean Tauzie, Yonghua Li, Tiina Ilmet, Elena Artimovich, Jillian Neal, Ted Hall, Sunil Parikh, Brian Kirmse, Patrick Jean-Philippe, Jingyang Chen, William R. Prescott, Paul Palumbo, Patrick E. Duffy, William Borkowsky, for the P1068s Study Team

**Affiliations:** 1 Batson Children’s Hospital, Department of Pediatrics (Division of Infectious Diseases) and Department of Microbiology, University of Mississippi Medical Center, Jackson, Mississippi;; 2 Laboratory of Malaria Immunology and Vaccinology, National Institute of Allergy and Infectious Diseases, National Institutes of Health, Rockville, Maryland;; 3 Department of Pediatrics, Division of Infectious Disease and Immunology, New York University School of Medicine, New York, New York;; 4 Biostatistics Research Branch, National Institute of Allergy and Infectious Diseases, National Institutes of Health, Rockville, Maryland;; 5 Kamuzu Central Hospital, University of North Carolina at Chapel Hill Lilongwe Project, Lilongwe, Malawi;; 6 Cornell Clinical Trials Unit, Weill Cornell Medicine, New York, New York;; 7 University of Maryland, Division of Malaria Research, Institute for Global Health, University of Maryland School of Medicine, Baltimore, Maryland;; 8 HYDAS World Health, Inc., Hummelstown, Pennsylvania;; 9 Yale Schools of Public Health and Medicine, New Haven, Connecticut;; 10 Department of Pediatrics, Division of Medical Genetics, University of Mississippi Medical Center, Batson Children’s Hospital, Jackson, Mississippi;; 11 HJF-DAIDS, Division of the Henry M. Jackson Foundation for the Advancement of Military Medicine, Inc., Contractor to NIAID, NIH, DHHS, Bethesda, Maryland;; 12 Ben Towne Center for Childhood Cancer Research, Seattle Children’s Research Institute, University of Washington, and Fred Hutchinson Cancer Research Center, Seattle, Washington;; 13 Division of Infectious Diseases and International Health, Geisel School of Medicine at Dartmouth, Lebanon, New Hampshire

## Abstract

Laboratory data and prior pediatric reports indicate that HIV protease inhibitor (PI)–based antiretroviral therapy (ARV) kills gametocytes and reduces rates of gametocytemia, but not asymptomatic parasitemia, in a high malaria-transmission area. To determine whether ARV regimen impacts these rates in areas with less-intense malaria transmission, we compared asymptomatic parasitemia and gametocytemia rates in HIV-infected children by ARV regimen in Lilongwe, Malawi, an area of low-to-moderate transmission intensity. HIV PI lopinavir–ritonavir (LPV–rtv) ARV– or non-nucleoside reverse transcriptase inhibitor nevirapine ARV–treated children did not differ in the rates of polymerase chain reaction-detected asymptomatic parasitemia (relative risk [RR] 0.43 95% confidence interval [CI] [0.16, 1.18], *P* value 0.10) or microscopically detected gametocytemia with LPV–rtv ARV during symptomatic malaria (RR 0.48 95% CI [0.22,1.04] *P* value 0.06). LPV–rtv ARV was not associated with reduced rates of asymptomatic parasitemia, or gametocytemia on days of symptomatic malaria episodes, in HIV-infected children. Larger studies should evaluate whether ARV impacts transmission.

## INTRODUCTION

HIV and malaria occur co-endemically in sub-Saharan Africa.^1^ Laboratory data show that HIV protease inhibitors (PIs) kill various life cycle stages of malaria parasites.^2–6^ PIs are second-line World Health Organization (WHO)-recommended antiretroviral therapy (ARV) for children above 3 years old and first-line ARV for those below 3 years.^7^ Clinical studies have shown that HIV-infected children on PI ARV may have a modest reduction in clinical malaria episodes, and the effect may be partially attributed to pharmacokinetic interactions resulting in an increase in antimalarial drug levels.^8–12^ In addition, laboratory data^4,5^ and recent pediatric clinical studies indicate that HIV PI lopinavir–ritonavir (LPV–rtv) ARV, when compared with non-nucleoside reverse transcriptase inhibitor (NNRTI) ARV, is associated with reduced gametocytemia,^11,13^ but not asymptomatic parasitemia,^13^ rates in high malaria-transmission areas.

Because malaria transmission intensity influences malaria infection and intervention efficacy, we evaluated the malaria impact of different ARV regimens in HIV-infected children by measuring asymptomatic parasitemia and gametocytemia in an area of low-to-moderate transmission. We recently reported an association between increased time to recurrent positive malaria blood smears in LPV–rtv ARV–treated subjects compared with nevirapine (NVP) ARV–treated subjects, when accounting for an LPV–rtv and antimalarial treatment interaction, in an observational pediatric study.^10^ Herein, we measure and compare rates of asymptomatic parasitemia and gametocytemia in children receiving differing ARV regimens.

## METHODS

### Study design.

The study was approved by site-specific institutional review boards; each child’s parent or legal guardian provided written informed consent.^10^ The study design was as previously described.^10,14^ The study was conducted at three sites with endemic-malaria transmission according to published data at the time, which included Kampala, Uganda; Lusaka, Zambia; and Lilongwe, Malawi; analysis was performed only on data from the Malawi site, however, because of low blood smear positivity rates at the other sites, as previously described.^14^ Briefly, subjects who enrolled in our study, P1068s, were HIV-infected children of age 2–36 months who qualified for treatment according to WHO criteria and were randomized to initiate PI- or NNRTI ARV in the larger HIV treatment study (P1060).^10,14^ Subjects received trimethoprim–sulfamethoxazole prophylaxis were given insecticide-treated bed nets, were breastfed, and lived within 30 km of the study site.^10^ Clinical illness (including malaria) was managed according to standard guidelines.^15,16^ Study visits occurred every 12 weeks and during intercurrent illness.^10^ Giemsa-stained thick smear and dried blood spots (DBS) were collected at each visit. Gametocytemia by smear was assessed by two microscopists, with a third microscopist who resolved discrepant results. Confirmatory polymerase chain reaction (PCR) was performed from DBS as previously described.^10^ Asymptomatic parasitemia was defined as parasite detection by PCR in the absence of a confirmed clinical malaria episode (CCM). CCM was defined as a positive blood smear with diagnosed malaria symptoms.^15^ Recrudescent infections (by microsatellite genotyping) were excluded. CD4% and HIV viral load were measured at study visits.

### Microsatellite genotyping.

To determine recrudescence, we measured expected heterozygosity in six *Plasmodium falciparum*–unlinked neutral loci (TA81, TA40, pfPK2, PolyA, TA87, ARA2). Microsatellites were amplified and analyzed using previously published methods.^17^ Fragment size was visualized using an Applied BioSystems 3730XLDNA sequencer. Electropherogram analysis was performed using Genemapper software (version 4.0; ABI). A Perl script was used to assign raw electropherogram scores to an integer allele size based on the expected repeat length and variation seen in the positive controls, and recrudescences were defined as previously described.^18^

### Statistics.

Statistical analysis was performed with R software, version 3.1.3. Negative binomial models were used for the count of PCR for asymptomatic parasitemia or gametocytemia rates per subject with an offset of time on treatment of entry into P1068 compared with the parent study P1060. Gametocytemia rates for CCM or non-CCM visits were compared regardless of the regimen, and then, gametocytemia rates were compared between treatment groups overall (at CCM and non-CCM visits), or at CCM or non-CCM visits, separately. Models were adjusted for gender, age at enrollment, baseline CD4, and time from enrollment in the parent study to the time of enrollment in the P1068s. A Wilcoxon signed rank test was used to compare gametocyte prevalence counts during confirmed clinical malaria or routine visits paired within the subject, regardless of the ARV regimen.

## RESULTS

Thirty-one children were enrolled between September 2009 and December 2011 from Kamuzu Central Hospital, Lilongwe, Malawi; demographic information and ARV regimens for these patients was been previously reported.^10^ Of 31 children, 18 started on the study on LPV–rtv ARV and 13 on NVP ARV. Eight patients who were randomized to start on NVP ARV switched to LPV–rtv ARV because of HIV treatment failure,^10^ with two subjects switching to LPV–rtv ARV before enrolling in the P1068s. One patient withdrew because of moving too far away from the study site to attend visits. We followed the enrolled children for a total of 20,771 person days on LPV–rtv and 9,911 person days on NVP ARV.

Between September 2009 and December 2011, 153 positive asymptomatic parasitemia episodes were identified. Microsatellite genotyping revealed two recrudescent infections (data not shown). Asymptomatic parasitemia and gametocytemia rates/person month were 0.03 and 0.05 while on LPV–rtv ARV, and 0.069 and 0.26 while on NVP ARV.

No significant difference in asymptomatic parasitemia rates in children on LPV–rtv ARV compared with NVP ARV were detected (RR 0.43 95% confidence interval [CI] [0.16, 1.18], *P* value 0.10). For overall gametocytemia rates, we found a significant difference between the number of gametocytemia detected during CCM and non-CCM visits when paired within individual (*P* value 0.02), regardless of the regimen. When comparing between the treatment groups, no significant difference was observed in overall gametocytemia rates (gametocytemia counted at both CCM and non-CCM visits) (RR 0.67 95% CI [0.39, 1.17] *P* value 0.16) when comparing LPV–rtv ARV with NVP ARV groups. Similarly, when comparing gametocytemia during CCM visits between LPV–rtv ARV or NVP ARV, children on LPV–rtv ARV did not have significant differences in gametocytemia rates, with concurrent CCM (RR 0.48 95% CI [0.22, 1.04] *P* value 0.06). Lastly, we detected no significant difference in gametocytemia rates with non-CCM visits (adjusted RR 1.01; 95% CI [0.33, 3.07]; *P* = 0.99) (Table 1).

**Table 1:** Summary of rates of asymptomatic parasitemia and gametocytemia for children on lopinavir–ritonavir antiretroviral therapy

	RR	Confidence interval	*P* value
Asymptomatic parasitemia	0.43	(0.16, 1.18)	0.10
Gametocytemia (overall, or CCM + non-CCM visits)	0.67	(0.39, 1.17)	0.16
Gametocytemia (non-CCM visits)	1.01	(0.33, 3.07)	0.99
Gametocytemia (during CCM visits)	0.48	(0.22, 1.04)	0.06

Adjusted for gender, age at enrollment, baseline CD4, and time from enrollment in the parent study to the time of enrollment in the P1068s. The indicator of PI-based ARV was based on having enrolled on the substudy while receiving PI-based ARV; two subjects had switched to PI-based ARV from their randomized treatment before entry into the substudy. ARV = antiretroviral therapy; CCM = confirmed clinical malaria; PI = protease inhibitor.

## DISCUSSION

In an area of low-to-moderate transmission, LPV–rtv ARV was not associated with reduced rates of asymptomatic parasitemia, or gametocytemia with or without concurrent symptomatic malaria episodes, in HIV-infected children.

Our previous study indicated that the reduced frequency of recurrent positive blood smears was only observed when accounting for a drug interaction between LPV–rtv ARV and the antimalarial (artemether–lumefantrine). In this report, however, we did not detect differences in asymptomatic parasitemia. Direct PI ARV killing of malaria parasites may not be significant, or our study may be underpowered, both because of the small size of the study and decreased likelihood of finding younger children with asymptomatic parasitemia in an area of low-to-moderate transmission.^19^ Indeed, the majority of infections being new rather than recrudescent may also reflect sampling which was performed mostly every 3 months, with the exception of intercurrent illness visits.

As expected, gametocytemia during CCM was more commonly detected when compared with non-CCM episodes.^20^ We compared the gametocyte prevalence overall between children on LPV–rtv ARV or NVP ARV but did not detect any significant difference between the groups when comparing overall (CCM and non-CCM) episodes. However, when limiting gametocytemia analysis to CCM visits, a significant difference was not appreciated.

A larger, randomized previous pediatric study that was conducted in an area of high-intensity malaria transmission also found that PIs were also not associated with reduced asymptomatic parasitemia in HIV-infected children, despite the study reporting fewer cases of recurrent clinical malaria with LPV–rtv ARV when compared with NNRTI ARV in an area of high malaria transmission intensity.^9^ This finding was partially attributed to a pharmacokinetic interaction between the ritonavir component of LPV–rtv and the antimalarial drugs, resulting in a prolonged period of lumefantrine detection,^9^ which is consistent with our prior publication.^10^ Moreover, analysis revealed no difference in gametocyte prevalence for children receiving LPV–rtv ARV compared with NNRTI ARV. However, when evaluating gametocytemia difference on the day of malaria diagnosis, they also found that it was much more likely that a child was gametocytemic on the day of malaria diagnosis, and within this analysis, LPV–rtv ARV was associated with significantly lower risk of gametocytemia.^13^ The data we report herein parallel some of these findings, except that gametocytemia on the day of CCM in LPV–rtv ARV compared with NVP ARV–treated children was not significantly different (*P* = 0.06). Part of this difference may be due to our study comparing children on LPV–rtv ARV with those on NVP ARV, whereas the prior study compared children on NNRTI ARV (either NVP or efavirenz, EFV) to those on LPV–rtv.^9^ This is of note as EFV has been shown to reduce antimalarial exposure much more significantly than NVP.^11^

PIs kill malaria gametocyte and transmission forms at clinically relevant levels through an unclear mechanism.^4,5,8^ Clinical trials from adult and pregnant women have shown little or no PI effect on clinical malaria, but pediatric data suggest that reduction of clinical malaria occurs with PI ARV, possibly because of direct parasite killing or pharmacokinetic effects.^4,8^ Our data suggest that HIV PI–based ARV did not reduce the asexual parasite pool because we found no difference in asymptomatic parasitemia rates. Lack of significant difference in gametocytemia rates between ARV groups similarly suggests a lack of PI-gametocytocidal effect.

A limitation of our study is our small sample size. Moreover, we were not able to assess gametocytemia differences at time points post treatment to account for residual drug interaction effects, although similar previous assessments resulted in no significant differences.^13^

A combination of interventions will likely eradicate malaria. Further studies are needed to evaluate whether PI ARV reduces gametocytemia and impacts transmission.
